# The Greenish Flower Phenotype of *Habenaria radiata* (Orchidaceae) Is Caused by a Mutation in the *SEPALLATA*-Like MADS-Box Gene *HrSEP-1*

**DOI:** 10.3389/fpls.2018.00831

**Published:** 2018-06-19

**Authors:** Mai Mitoma, Akira Kanno

**Affiliations:** Graduate School of Life Sciences, Tohoku University, Sendai, Japan

**Keywords:** greenish flower, floral homeotic mutant, *SEPALLATA*-like gene, MADS-box gene, retrotransposon, Orchidaceae

## Abstract

In *Arabidopsis thaliana*, the E-class *SEPALLATA* (*SEP*) genes are generally expressed across all floral whorls. These genes play fundamental roles in floral organ fate determination during development by interacting with other MADS-box gene products, such as those from A-, B-, and C-class genes. However, the function of *SEP* genes in orchid remains obscure. Here, we analyzed a mutant orchid cultivar with greenish flowers in *Habenaria radiata* and found that this phenotype is caused by the absence of SEP function. Wild type *H. radiata* flowers contain a column and two perianth whorls consisting of three greenish sepals, two white petals, and a lip (labellum). By contrast, the flowers of *H. radiata* cultivar ‘Ryokusei’ appear greenish, with three normal sepals in whorl 1, two greenish petals and a lip in whorl 2, and several sepaloid organs and a ventral column in whorls 3 and 4. We isolated two *SEP*-like genes (*HrSEP-1* and *HrSEP-2*) and two *AGAMOUS*-like genes (*HrAG-1* and *HrAG-2*) from wild type *H. radiata* and compared their expression in the wild type vs. the mutant cultivar. *HrAG-1* and *HrAG-2* were expressed in the column in the wild type, whereas these genes were expressed in the ventral column and in sepaloid organs that had been converted from a column in ‘Ryokusei.’ *HrSEP-1* and *HrSEP-2* were expressed in all floral organs in the wild type. However, in the mutant cultivar, *HrSEP-2* was expressed in all floral organs, while *HrSEP-1* expression was not detected. Thus, we analyzed the genomic structures of *HrSEP-1* in the wild type and ‘Ryokusei’ and identified a retrotransposon-like element in its first exon in ‘Ryokusei.’ Yeast two-hybrid assays demonstrated that HrSEP-1 interacts with HrDEF, HrAG-1, and HrAG-2. These results indicate that the mutant phenotype of ‘Ryokusei’ flowers is caused by the loss of function of *HrSEP-1*. Therefore, this gene plays an important role in column, lip, and petal development in *H. radiata* flowers.

## Introduction

The ABC model of floral organ identity determination was established based on genetic studies in *Arabidopsis thaliana* and *Antirrhinum majus* (Carpenter and Coen, 1990; Bowman et al., 1991; Schwarz-Sommer et al., 1992). According to this model, the activity of A-, B-, and C-class genes, alone or in combination, specifies the formation of the distinct organs of the four floral whorls. The A function specifies sepal formation in whorl 1, co-expression of the A and B functions specifies petal formation in whorl 2, B and C genes together determine stamen development in whorl 3, and C genes specify carpel development in whorl 4 (Coen and Meyerowitz, 1991; Weigel and Meyerowitz, 1994). The A-class MADS-box gene *APETALA1* (*AP1*)-like is required for the establishment of floral meristem and for specifying sepal and petal identity. The B-class floral homeotic genes, which are responsible for specifying petal and stamen identity, form two major clades: *DEFICIENS* (*DEF*)- and *GLOBOSA*-like genes. The C-class *AGAMOUS* (*AG*) genes play a central role in stamen and carpel development (Yanofsky et al., 1990).

*SEPALLATA* (*SEP*) genes are E-class MADS-box genes. SEP proteins form higher-order complexes together with A-, B-, and C-class gene products. *A. thaliana* contains four *SEP* genes: *SEP1, SEP2, SEP3*, and *SEP4*. The flowers of a triple mutant of three *SEP* genes (*sep1/sep2/sep3*) consist entirely of sepal-like organs (Pelaz et al., 2000), whereas in *sep1 sep2 sep3 sep4* quadruple mutants, all floral organs are converted into leaf-like organs (Ditta et al., 2004). Therefore, the four *A. thaliana* *SEP* genes function redundantly and are important for the activities of B- and C-function genes in petal, stamen, and carpel development (Honma and Goto, 2001; Theissen and Saedler, 2001). *SEP*-like genes have been isolated from many dicots, such as petunia (Ferrario et al., 2003; Vandenbussche et al., 2003; Matsubara et al., 2008) and tomato (Pnueli et al., 1991; Ampomah-Dwamena et al., 2002), as well as monocots such as rice (Arora et al., 2007) and maize (Lid et al., 2004). Phylogenetic analyses suggested that *SEP*-like genes are monophyletic and that they form two subclades, the SEP1/2/4-like clade (AGL2/3/4 clade) and the SEP3-like clade (AGL9 clade) (Zahn et al., 2005). Functional analyses of *SEP* orthologs have been conducted in several plants by disrupting RNA expression. The petunia *fbp2* mutant (a *SEP3* ortholog) exhibits greenish petals and ectopic inflorescences originating from the third floral whorl, while the *fbp5* mutant (a *SEP1/2* ortholog) exhibits no significant morphological changes (Vandenbussche et al., 2003). Double mutants of these two genes show significant changes, with the conversion of floral organs to leaf-like organs (Vandenbussche et al., 2003). In the monocot rice, simultaneous knockdown of the four *SEP*-like genes (*OsMADS1, OsMADS5, OsMADS7*, and *OsMADS8*) caused the transformation of all floral organs except the lemma into leaf-like structures. These findings indicate that the *SEP* orthologs are required for petal, stamen, and carpel formation in both dicots and monocots (Cui et al., 2010).

Orchidaceae is the largest family of flowering plants. Many orchids are highly valued for their elaborate flowers and unique organ structures. The floral organs of many Orchidaceae plants comprise three sepals in whorl 1, two petals and a lip in whorl 2, and a column (fused stamen and carpel) in whorls 3 and 4. Orchid B-, C/D-, and E-class MADS-box genes have been characterized, and gene duplications in each group have been analyzed (Tsai and Chen, 2006; Aceto and Gaudio, 2011; Mondragón-Palomino, 2013). To date, several *SEP*-like genes have been identified from a few orchid species, including *AdOM1* in *Aranda* (Lu et al., 1993), *DOMADS1* and *DOMADS3* in *Dendrobium* grex Madame Thong-In (Yu and Goh, 2000), *DcOSEP-1* in *Dendrobium crumenatum* (Xu et al., 2006), *OMADS6* and *OMADS11* in *Oncidium* Gower Ramsey (Chang et al., 2009), *PeSEP1-4* in *Phalaenopsis equestris* (Pan et al., 2014) and *CgSEP1-4* in *Cymbidium goeringii* Rchb.f (Xiang et al., 2017). Expression analysis of orchid *SEP*-like genes showed that *SEP3* orthologs (*DOMADS1, DcOSEP1, PeSEP1, PeSEP3*, and *OMADS6*) are expressed in sepals, petals, lips, and columns during flower development, whereas *CgSEP1, CgSEP3*, and *AdOM1* are rarely expressed in columns (Lu et al., 1993; Xiang et al., 2017). By contrast, the expression patterns of *SEP1/2* orthologs (*DOMADS3, PeSEP2, PeSEP4, OMADS11, CgSEP2*, and *CgSEP4*) vary among species. For example, in *Phalaenopsis, PeSEP2* is expressed in all floral organs, whereas *PeSEP4* is expressed at extremely low levels in all floral organs (Pan et al., 2014).

Studies involving ectopic expression in *Arabidopsis* and virus-induced gene silencing (VIGS) in *Phalaenopsis* were carried out to investigate the functions of *Phalaenopsis* SEPs in determining floral organ identity (Pan et al., 2014). Transgenic *35S:PeSEP3* *Arabidopsis* plants exhibited early flowering and much smaller flowers than the wild type, whereas transgenic *35S:PeSEP1* *Arabidopsis* plants showed no phenotypic changes compared with the wild type. The flowers of *PeSEP3-* and *PeSEP2/3-*silenced plants (obtained by VIGS) showed partial leaf-like structures in whorls 1 and 2, and epidermis identity as well as anthocyanin and chlorophyll contents were altered in these flowers. Although the functions of orchid *SEP*-like genes have been investigated via heterogenic transformation and VIGS, *sep* mutants have thus far not been identified in orchid. Such mutants would be very useful for analyzing the functions of *SEP* genes in orchid.

The genus *Habenaria* contains approximately 800 species, representing one of the largest genera in Orchidaceae (Yokota, 1990). *Habenaria* species are distributed throughout the world, with the highest concentrations in the tropical regions of Africa and Southeast Asia (Yokota, 1990). *H. radiata* is one of the most famous orchids in Japan. This small terrestrial orchid lives in wetlands in East Asia. *H. radiata* flowers have greenish sepals in whorl 1, two white petals and a lip in whorl 2, and a column in whorls 3 and 4. There are some mutant cultivars of *H. radiata*. One of these cultivars, ‘Hishou,’ has flowers with a white petaloid organ instead of a greenish dorsal sepal and two greenish lateral sepals replacing the lip-like organs (Kim et al., 2007).

We previously suggested that the floral phenotypes of ‘Hishou’ appear to be caused by the expanded expression of the *DEF-*like gene, *HrDEF*, which belongs to DEF clade 3 (Kim et al., 2007). In the current study, we characterized a mutant cultivar of *H. radiata* named ‘Ryokusei.’ This cultivar has greenish flowers, and its column has been converted into greenish sepaloid organs. We isolated C- and E-class genes in wild type *H. radiata* and compared the expression of these genes in the wild type and ‘Ryokusei,’ finding that the expression of the *SEP*-like gene, *HrSEP-1*, was suppressed in ‘Ryokusei’. We compared the genomic structures of *HrSEP-1* in the wild type and ‘Ryokusei’ and found that this mutant character is caused by the insertion of a retrotransposon in *HrSEP-1*. Our findings suggest that *HrSEP-1* plays an important role in floral development in *H. radiata*.

## Materials and Methods

### Plant Materials

*Habenaria radiata* ‘Aoba’ (the wild type) and ‘Ryokusei’ (a mutant with greenish flowers) were used in this study. These cultivars were obtained from a garden shop and cultivated in a greenhouse at the Graduate School of Life Sciences, Tohoku University, Japan. Floral buds (0.7–1.0 cm) were collected and stored at -80°C for subsequent RNA extraction. Sepals, petals, and columns (fused stamens and carpel) were dissected from 5 to 10 flowers and used for expression analysis.

### Isolation of MADS-Box Genes From *H. radiata*

Total RNA was prepared from whole floral buds of ‘Aoba’ using an RNeasy Plant Mini Kit (QIAGEN). Poly A-tailed mRNA was separated from total RNA using a Dynabeads mRNA Purification Kit (Life Technologies). cDNA was synthesized from mRNA using oligo dT primer P019HA with AMV Reverse Transcriptase following the manufacturer’s instructions (Roche). cDNA fragments were isolated from MADS-box genes by 3′ rapid amplification of cDNA ends (RACE) using MADS-box-specific degenerate primers alongside a species-specific adaptor primer. The PCR products were electrophoresed on agarose gels, extracted using a QIAquick Gel Extraction Kit (QIAGEN), and cloned into pGEM-T Easy Vector (Promega). Upstream sequences of the MADS-box genes were isolated by 5′RACE using a 5′/3′RACE Kit, 2nd Generation (Roche), according to the manufacturer’s protocol. The primers used in this study are shown in **Supplementary Table S1**. The sequences of the HrAGs and HrSEPs isolated in this study were submitted to GenBank under accession numbers LC369631–LC369634.

### Phylogenetic Analysis of *HrAGs* and *HrSEPs*

Phylogenetic analysis was conducted using MEGA v7.0.26 software (Kumar et al., 2016). Predicted amino acid sequences of known MADS-box genes were obtained from the EMBL/DDBJ/GenBank DNA database (**Supplementary Tables S2, S3**). Full-length amino acid sequences were used to construct the phylogenetic trees and aligned using Clustal W. JTT + G was selected and used to construct a neighbor-joining tree, with 1000 bootstrap replicates. In the case of AG-like genes, non-conserved C-regions were excluded.

### Expression Analysis via Semi-Quantitative and Quantitative RT-PCR

Semi-quantitative reverse-transcription PCR (semi-qRT-PCR) and quantitative RT-PCR (RT-qPCR) were performed to examine the expression levels of the *DEF, AG*, and *SEP* genes. Total RNA was extracted from sepals, petals, lips, and columns (‘Aoba’) or sepaloid organs converted from dorsal and ventral columns (‘Ryokusei’). cDNA was synthesized as described above, using oligo dT primers P019HA and P019HR for ‘Aoba’ and ‘Ryokusei,’ respectively. RT-qPCR was conducted using a MiniOpticon Real-time PCR Detection System with CFX Manager software (Bio-Rad). The cycling program was as follows: one cycle at 95°C for 3 min followed by 40 cycles of 95°C (10 s) and 64°C (1 min), with plate reading after each cycle. Gene-specific primers were designed for *HrDEF, HrSEP-1, HrSEP-2, HrAG-1*, and *HrAG-2* (**Supplementary Table S1**). The transcript levels of these five genes were determined using three experimental replicate PCRs for each cDNA sample. The *eEF1A* (*eukaryotic translation elongation factor 1A*) gene was used as an internal control for standardization.

### Isolation of the *HrSEP-1* Promoter From the Wild Type and ‘Ryokusei’ by DNA Walking

Genomic DNA was isolated from *H. radiata* leaves using a modified CTAB (hexadecyl trimethyl-ammonium bromide) method (Porebski et al., 1997). The genomic DNA was digested at 37°C overnight with four different blunt-end restriction enzymes (DraI, EcoRV, PvuII, and StuI). The digested DNA was ligated to a custom-designed adaptor from a Genome Walker kit at 16°C overnight. The constructed libraries were used as templates for two-step PCR. The primary PCR was performed using the outer adaptor primer (AP1) provided in the kit and a specific primer for *HrSEP-1* (GSP1). The secondary PCR was performed using the nested adaptor primer (AP2) and a nested specific primer for *HrSEP-1* (GSP2), using the primary PCR products as template. The secondary PCR products were cloned and sequenced as described above.

### Identification of Insertion Sequence in *HrSEP-1* Gene

Genomic DNAs were extracted from the leaves of wild type and ‘Ryokusei’ as described above. Transposon PCR amplifications were performed on leaf genomic DNA of ‘Ryokusei,’ using primers to specifically amplify between promoter and exon 1 of *HrSEP-1* (**Supplementary Table S1**). Purified PCR product was further sequenced. Retrotransposon-like insertion isolated in this study, designated as *Hret1*, were submitted to GenBank under accession numbers LC382365.

To confirm the insertion of retrotransposon-like structure, we performed genomic PCR. We used genomic DNA from the leaves of wild type and ‘Ryokusei’ after adjusting the concentration as 100 ng/μl. PCR was conducted in a 25 μL reaction mixture containing 50 ng total DNA, Tks Gflex DNA Polymerase (TaKaRa Bio Inc.), using the primers (50 pmol of each primer) P1 + P4 and P2 + P3, which are specific for the gene and retrotransposon, respectively. PCR amplification was performed in a TaKaRa PCR Thermal Cycler Dice (TaKaRa Bio Inc.). The following PCR cycling condition was used: denaturation for 1 min at 94°C, followed by 30 cycles of 10 s at 98°C, 15 s at 62°C, and 3 min at 68°C.

### Yeast Two-Hybrid Assays

Yeast two-hybrid analysis to investigate protein–protein interactions among B-class (HrDEF), C-class (HrAGs), and E-class (HrSEPs) proteins was performed using the GAL4 system. Full-length cDNA from HrDEF, HrSEP-1, and the two HrAGs was amplified by PCR using primers containing the attB sequence site to facilitate full-length cDNA cloning, whereas for HrSEP-2, the C-region was deleted to prevent autoactivation. The PCR fragments were ligated into both the prey vector pDEST22 and the bait vector pDEST32 using the Gateway system (Life Technologies). Bait and prey constructs were transformed into yeast strains PJ69-4α and PJ69-4A, respectively, using the lithium acetate method. The yeast transformations were screened on selection medium according to the manufacturer’s instructions (de Folter and Immink, 2011). To test for autoactivation, all PJ69-4α strains were plated on SD/-Leu -Trp -His medium, and 5 mM 3-amino-1,2,4-triazole (3-AT) was added to suppress background signals due to autoactivation. All interaction experiments were conducted in triplicate, and yeast growth was scored after 7 days of incubation at 20°C.

## Results

### Floral Morphology of ‘Aoba’ (Wild Type) and ‘Ryokusei’ (Greenish Flower Mutant)

**FIGURE 1:**
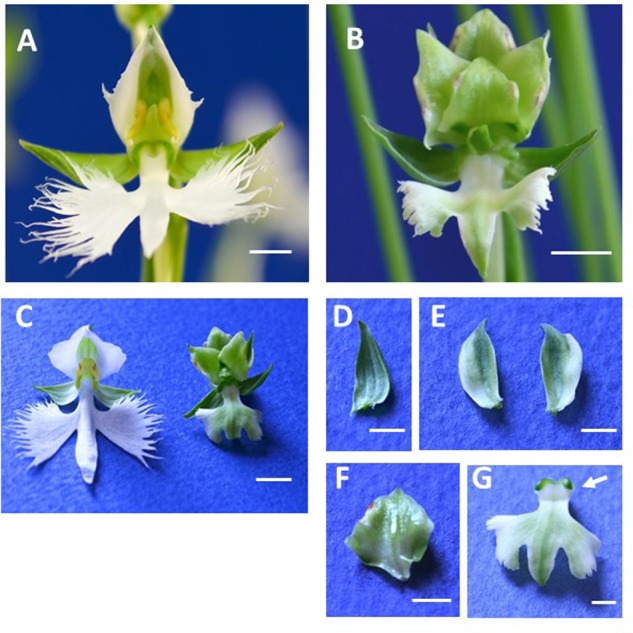
Flower morphology of *Habenaria radiata* ‘Aoba’ (wild type) and ‘Ryokusei’ (greenish flower mutant). **(A)** Wild type flower. The flower has three greenish sepals in whorl 1, two white petals and one white lip in whorl 2, and a column in whorls 3 and 4. **(B)** Flower of the greenish flower mutant ‘Ryokusei.’ The mutant has three normal sepals in whorl 1, two greenish petals and a lip in whorl 2, and sepaloid organs and a ventral column in whorls 3 and 4. **(C)** Wild type and ‘Ryokusei’ flowers before dissection. **(D–G)** Dissected ‘Ryokusei’ flower, with a sepal **(D)**, petals **(E)**, a sepaloid organ instead of a dorsal column **(F)**, and a greenish lip with a ventral column **(G)**. Arrow indicates the stigma-like structure **(G)**. Scale bars: 0.5 cm **(A–C)** and 0.25 cm **(D–G)**.

Wild type ‘Aoba’ flowers contain three greenish sepals in whorl 1, two white petals and a white lip in whorl 2, and a column in whorls 3 and 4 (**Figure 1A**). By contrast, ‘Ryokusei’ flowers appear greenish overall and are smaller than wild type flowers (**Figures 1B,C**). In the ‘Ryokusei’ perianth, two petals have been converted into greenish sepaloid structures and the lip is greenish and smaller than that of the wild type, although there are no notable differences in the three (greenish) sepals between flowers (**Figures 1D–G**). In whorls 3 and 4, the dorsal column (stamen-like organ) has been converted into several sepaloid organs, with degenerated pollinium-like organs on the edges of these organs (**Figures 1B,F**). The form of the ventral column (carpel-like organ) is almost the same as that of the wild type, and ‘Ryokusei’ flowers contain stigma-like structures, although this cultivar is sterile (**Figure 1G**).

‘Ryokusei’ was derived from a *H. radiata* strain found in Shodoshima Island, Japan (Mitsuhashi, 1988). Since the floral morphology of the wild type and ‘Ryokusei’ is very different, we analyzed the sequences of the nuclear ribosomal internal transcribed spacer (ITS) regions from ‘Ryokusei’ and various *Habenaria* species to confirm the origin of this mutant cultivar (**Supplementary Figure S1**). We designed a primer set based on the ITS region and amplified this region in ‘Aoba’ and ‘Ryokusei.’ We obtained PCR products of approximately 500 bp and determined the sequences of the ITS regions. We compared the ITS sequence of ‘Ryokusei’ with that of other *Habenaria* species (*H. propinquior, H. arenaria, H. laevigata, H. clavata*, and *H. lithophila*), finding that the ITS sequence of ‘Ryokusei’ is completely identical to that of *H. radiata* ‘Aoba.’ Phylogenetic analysis confirmed that ‘Ryokusei’ is classified in *H. radiata* (**Supplementary Figure S1**).

### cDNA Cloning of *AG-* and *SEP-*Like Genes From *H. radiata*

**FIGURE 2:**
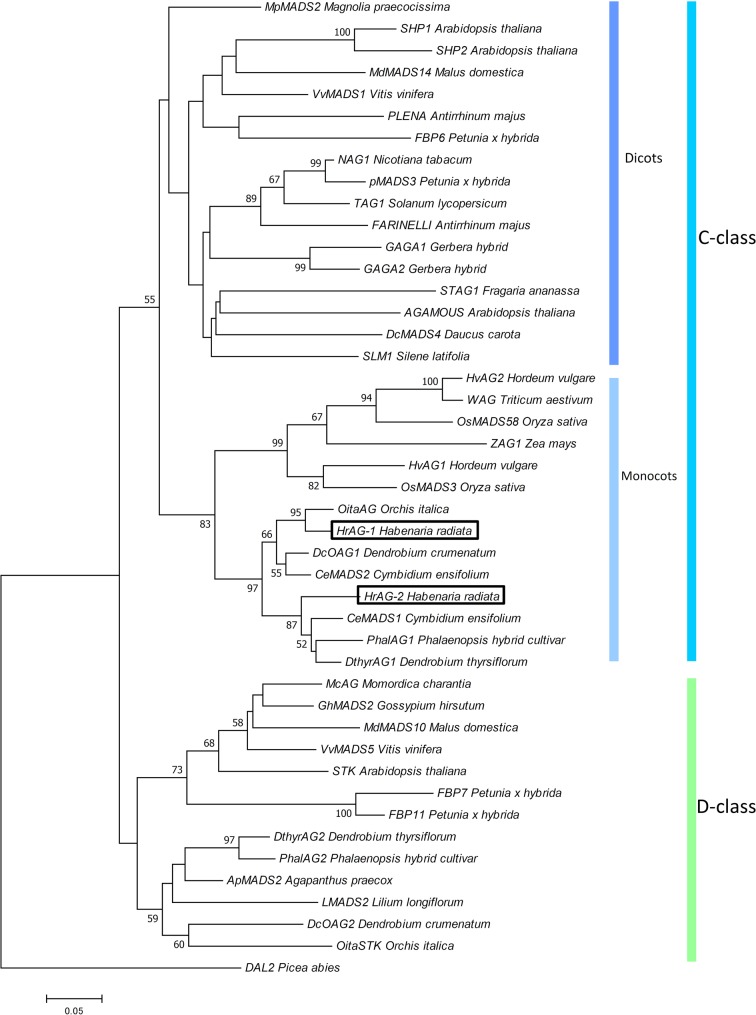
Phylogenetic analysis of AG-like genes. The phylogenetic tree was constructed using the neighbor-joining method with the JTT + G model. The genes isolated from *H. radiata* are boxed. Bootstrap values greater than 50% from 1000 replicates are shown on the nodes.

**FIGURE 3:**
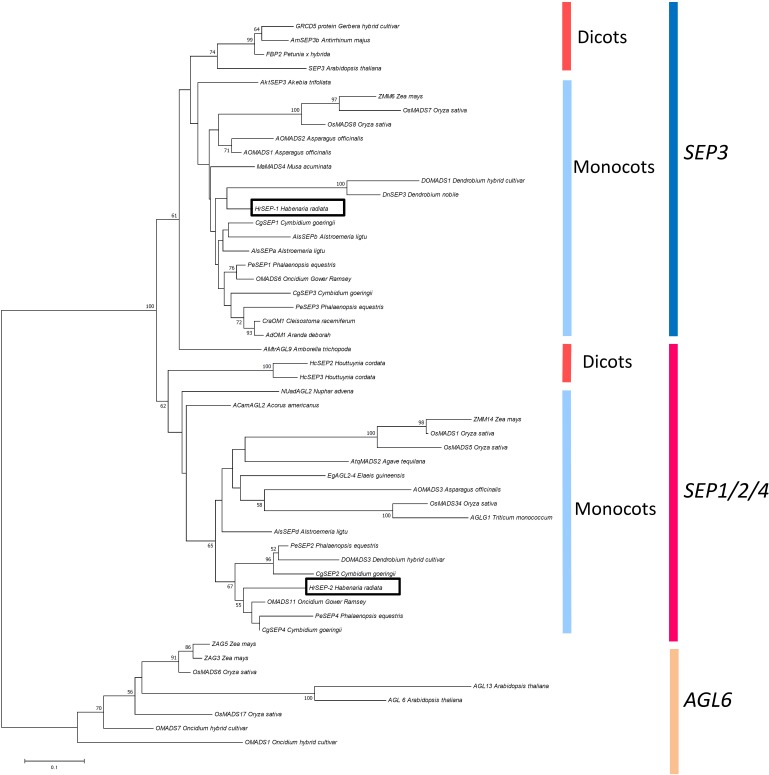
Phylogenetic analysis of SEP-like genes. The phylogenetic tree was constructed using the neighbor-joining method with the JTT + G model. The genes isolated from *H. radiata* are boxed. Bootstrap values greater than 50% from 1000 replicates are shown on the nodes.

**FIGURE 4:**
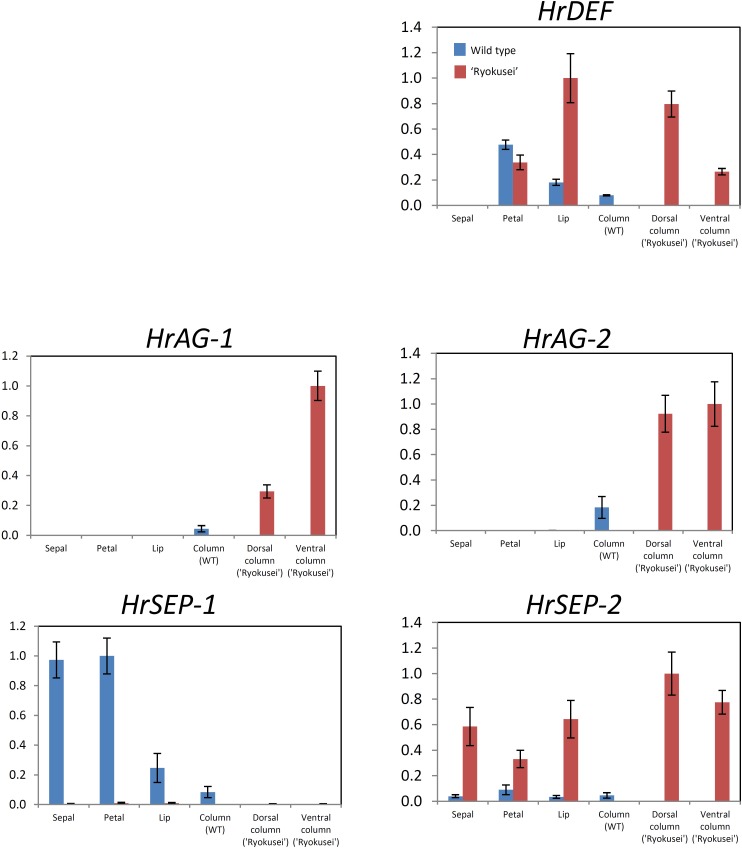
Quantitative RT-PCR analysis of MADS-box genes in sepal, petal, lip, and column tissue from wild type *H. radiata* flowers and sepal, greenish petal, greenish lip, and sepaloid tissue in whorls 3 and 4 (dorsal and ventral column) from ‘Ryokusei.’ Error bars represent the standard error from three experimental replicates.

We isolated four cDNA clones of MADS-box genes from *H. radiata* ‘Aoba’ by RACE using MADS-box-specific degenerate primers. After cloning and sequencing the cDNA fragments, BLAST searches (TBLASTN) revealed that two of these clones shared high sequence similarity with *SEP*-like genes, and two were highly similar to *AG*-like genes. After obtaining the 5′-regions of the cDNAs by 5′RACE, we isolated full-length cDNA clones by PCR using gene-specific primers. Phylogenetic analysis classified the four genes into two major groups according to gene lineage: the SEP-like and AG-like clades (**Figures 2–4**).

We designated the two *AG*-like genes from wild type *H. radiata* as *HrAG-1* and *HrAG-2* (**Figure 2**). The 687 bp full-length *HrAG-1* cDNA encodes a 228 aa protein, and the 702 bp full-length *HrAG-2* cDNA encodes a 233 aa protein. These genes consist of a MADS-box domain, an I-region, a K-domain, and a C-region. In addition, both HrAG-1 and HrAG-2 contain AG motifs I and II at their C-terminal ends. HrAG-1 shares 83.8% identity with HrAG-2 on the amino acid level. HrAG-1 and HrAG-2 share high homology with the *Phalaenopsis* protein, PhalAG1 (93 and 91% identity, respectively).

The two *SEP*-like genes from *H. radiata* were designated *HrSEP-1* and *HrSEP-2* (**Figure 3**). The 657 bp full-length *HrSEP-1* cDNA encodes a 218 aa protein, and the 735 bp full-length *HrSEP-2* cDNA encodes a 244 aa protein. HrSEP-1 and HrSEP-2 contain a MADS-domain, an I-region, and a K-domain. Although HrSEP-2 has SEP-I and SEP-II motifs at its C-terminal region, the C-terminal region of HrSEP-1 lacks a SEP-II motif. The deduced amino acid sequences of HrSEP-1 and HrSEP-2 share 58.8% identity. A comparison of published SEP amino acid sequences with that of HrSEP-1 showed that it shares 88% identity with DOSEP1 from *Dendrobium* and 87% identity with PeSEP 1 from *Phalaenopsis*. HrSEP-2 shares high homology (79% sequence identity) with DOMADS3 from *Dendrobium* and 81% identity with PeSEP 2 from *Phalaenopsis*.

### Expression Analysis of *DEF-, AG-*, and *SEP-*Like Genes From *H. radiata*

We performed semi-quantitative and quantitative RT-PCR to analyze the expression patterns of the *DEF*-like gene (*HrDEF*), two *SEP*-like genes (*HrSEP-1, HrSEP-2*), and two *AG*-like genes (*HrAG-1, HrAG-2*) using dissected floral organs from wild type (sepal, petal, lip, and column) and ‘Ryokusei’ (sepal, petal, lip, and dorsal and ventral columns) in *H. radiata* (**Supplementary Figure S2 and Figure 4**). These experiments were repeated three times, with each replicate revealing the same expression patterns.

In the wild type, *HrDEF* was expressed in petals, lips, and columns, whereas *HrDEF* transcript was not detected in sepals. The expression patterns of *HrDEF* in the wild type were consistent with those of other orchid species and generally fit the ‘orchid code’ for organ identity in the orchid perianth. In ‘Ryokusei,’ *HrDEF* expression was detected in the petal and lip, as well as the dorsal and ventral column. In ‘Ryokusei,’ the expression level of this gene was higher in lip and dorsal and ventral column tissue and lower in petal tissue compared with the wild type. These results indicate that the expression patterns of *HrDEF* differed in the wild type vs. ‘Ryokusei.’

In the wild type, *HrAG-1* and *HrAG-2* were expressed only in columns, which matches the expression patterns of their orthologs in other orchid species. In ‘Ryokusei,’ these genes were expressed only in dorsal and ventral columns, as in the wild type. However, *HrAG-1* and *HrAG-2* were expressed at higher levels in the dorsal and ventral columns of ‘Ryokusei’ compared with wild type columns. In addition, *HrAG-1* was expressed at higher levels in the ventral vs. the dorsal column. Thus, the expression patterns of *HrAG-1* and *HrAG-2* in the wild type and ‘Ryokusei’ were similar, whereas the expression level of these genes differed strongly among lines.

*HrSEP-1* and *HrSEP-2* transcripts were widely detected throughout all floral organs in the wild type. However, in ‘Ryokusei,’ *HrSEP-2* was strongly upregulated in all floral organs (**Supplementary Figure S2 and Figure 4**), whereas *HrSEP-1* expression was significantly suppressed in all floral organs. These results suggest that the suppressed expression of *HrSEP-1* in ‘Ryokusei’ might be the cause of greenish flower formation in this cultivar.

### A Retrotransposon Insertion Is Detected in the First Exon of *HrSEP-1* in ‘Ryokusei’

**FIGURE 5:**
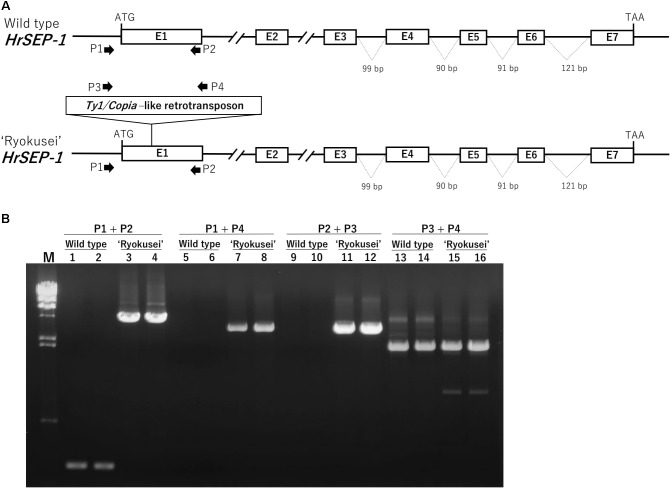
Genomic structure of *HrSEP-1* from wild type and ‘Ryokusei’ in *H. radiata*. **(A)** Schematic diagrams of the genomic structures of *HrSEP-1* from the wild type and ‘Ryokusei.’ White boxes indicate exons; the ATG start codons and TAA stop codons are also shown. ‘Ryokusei’ has a *Ty1/Copia*-like retrotransposon in the first exon of *HrSEP-1*. **(B)** PCR analysis of *HrSEP-1* from the wild type and ‘Ryokusei.’ PCR was performed using primer sets specific for *HrSEP-1* gene (P1 and P2) and retrotransposon (P3 and P4), as shown in **(A)**.

As mentioned above, *HrSEP-1* expression was strongly suppressed in all floral organs of ‘Ryokusei.’ To clarify the molecular mechanism involving the suppressed expression of this gene, we compared the gene structures of *HrSEP-1* in the wild type vs. ‘Ryokusei.’ In both plants, *HrSEP-1* consists of seven exons and six introns. The size of each exon is identical in both plants, except the first exon (**Figure 5A**). We also isolated the promoter region of *HrSEP-1* by Genome Walker PCR and detected 140 bp promoter regions with identical sequences in the wild type and ‘Ryokusei.’ To confirm the structure of the first exon of *HrSEP-1* in the wild type and ‘Ryokusei,’ we performed PCR amplification with primers designed based on the sequence of promoter region and first exon (P1 and P2 primers; **Figure 5B**). The P1 and P2 primer pair amplified a PCR product of approximately 250 bp from the wild type, whereas the size of the PCR product from ‘Ryokusei’ was approximately 4,800 bp (**Figure 5B**). We cloned and sequenced these PCR products and found that the first exon of *HrSEP-1* from ‘Ryokusei’ contained an insertion sequence. This insertion sequence has typical features of a LTR-retrotransposon, and was therefore designated *Hret1* (*Habenaria* retrotransposon 1). *Hret1* is 4,534 bp long, with 5 bp of target site duplication (TSD) [213 bp of a long terminal repeat (LTR) – 407 bp of group-specific antigen (GAG) – 284 bp of integrase (IN) – 728 bp of reverse transcriptase (RT) – 425 bp of ribonuclease H (RH) – 213 bp of LTR] – 5 bp of TSD, indicating that *Hret1* is a *Ty1/Copia-*type retrotransposon. *Hret1* is inserted in the first exon with reverse orientation to *Hret1* transcript. To confirm the insertion of *Hret1* in the first exon of *HrSEP-1* gene in ‘Ryokusei,’ we performed PCR amplification with primers specific for the gene and retrotransposon. Using the primers of P1 + P4 and P2 + P3, which are specific for the gene and retrotransposon, respectively, DNA fragment was amplified in ‘Ryokusei,’ but not in wild type. This data clearly showed the insertion of retrotransposon in *HrSEP-1* gene of ‘Ryokusei.’ We obtained a PCR product of approximately 2000 bp with P3 and P4 primer pair from both wild type and ‘Ryokusei,’ indicating that *Hret1* exist in wild type plant as well as ‘Ryokusei’ cultivar.

### Protein–Protein Interactions of HrSEP-1 With Other MADS-Box Proteins in *H. radiata*

**FIGURE 6:**
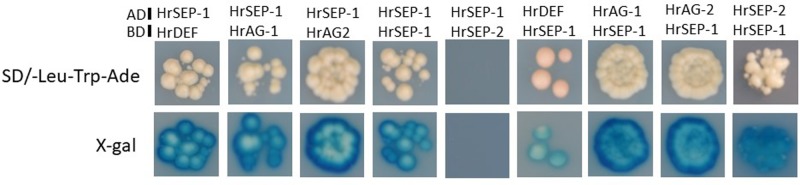
Analysis of protein-protein interactions between HrSEP-1 and other MADS-box proteins in *H. radiata* by GAL4 yeast two-hybrid analysis. Baits were expressed as GAL4 DNA-BD fusion proteins in pDEST32 and transformed into the PJ69-4α yeast strain, and preys were expressed as GAL4 AD fusion proteins in pDEST22 and transformed into the PJ69-4A yeast strain. Activation of ADE was determined on SD selection medium (SD/-Leu-Trp-Ade), and activation of *lacZ* is indicated by *X*-gal staining. Full-length amino acid sequences were used in this study, except for HrSEP-2; to avoid autoactivation, the C-region was removed from HrSEP-2.

To investigate whether HrSEP-1 forms protein complexes with other MADS-box proteins, we performed a GAL4-based yeast two-hybrid assay. Specifically, we assessed protein complex formation with HrSEP-1 in a GAL4-based yeast two-hybrid assay for HrDEF, two AG proteins (HrAG-1 and HrAG-2), and one SEP protein (HrSEP-2). Autoactivation tests revealed no background signals for yeast strains containing bait only (data not shown), except for HrSEP-2. For HrSEP-2, we used truncated HrSEP-2 protein containing the MADS-domain, I-region, and K-domain. HrSEP-1 protein interacted with B-class HrDEF protein and C-class HrAG-1, HrAG-2, and HrSEP-1 proteins. As shown in **Figure 6**, heterodimerization of HrSEP-1/HrDEF, HrSEP-1/HrAG-1, and HrSEP-1/HrAG-2 was detected even when the bait and prey constructs were switched. A HrSEP-1/HrSEP-2 interaction was detected when HrSEP-1 was used as bait, whereas this interaction was not detected when HrSEP-1 was used as prey, suggesting it is likely that HrSEP-1/HrSEP-2 interact weakly. These results suggest that heterodimers of HrSEP-1/HrDEF, HrSEP-1/HrAG-1, and HrSEP-1/HrAG-2 bind stably to each other, unlike HrSEP-1/HrSEP-2 (**Figure 6**).

## Discussion

### A Retrotransposon Insertion Is Present in *HrSEP-1* in ‘Ryokusei’

In this study, we investigated the cause of greenish flower formation in the mutant *H. radiata* cultivar ‘Ryokusei.’ Instead of the white flowers of the wild type, ‘Ryokusei’ flowers have an overall greenish color. Although the petals, lip, and column have been converted into sepaloid structures in this mutant orchid, the sepals in whorl 1 are unaffected. The dorsal column has also been converted into sepaloid organs that are thick and partially yellow at both ends. In addition, the number of sepaloid organs derived from the column varies from three to five. The floral morphology of ‘Ryokusei’ appears to resemble that of indeterminate flowers, suggesting that this phenotype is caused by a mutation in an *AG*-like gene. Thus, we isolated two *AG*-like genes (*HrAG-1* and *HrAG-2*) from *H. radiata* (**Figure 4**). However, these genes were highly expressed in the column-derived, greenish sepaloid structures in ‘Ryokusei’ (**Figure 4**). These results suggest that the floral phenotype of ‘Ryokusei’ is not caused by the mutation of *AG*-like genes.

The morphological changes in *Arabidopsis* *ag* mutants are restricted to male and female reproductive organs (Bowman et al., 1989), whereas in ‘Ryokusei,’ we also detected morphogenetic changes in the lip and petal in whorl 2, as well as the column in whorls 3 and 4 (**Figure 1**). This phenotype is similar to that of a rice *OsMADS7/8* knockdown line (Cui et al., 2010): the knockdown of *OsMADS7* and *OsMADS8* in rice led to significant morphological changes in the organs of the three innermost whorls (Cui et al., 2010). Thus, we isolated two *SEP*-like genes (*HrSEP-1* and *HrSEP-2*) from wild type *H. radiata* and analyzed their expression patterns. These genes were expressed in all floral organs of the wild type, matching the expression patterns of other orchid *SEP* genes from *Cymbidium, Oncidium*, and *Phalaenopsis* (Chang et al., 2009; Pan et al., 2014; Xiang et al., 2017). By contrast, *HrSEP-1* transcript was not detected in ‘Ryokusei’, although *HrSEP-2* was expressed in all floral organs (**Figure 4**). These results suggested that a deletion or structural rearrangement might have occurred in *HrSEP-1* in ‘Ryokusei.’ Thus, we isolated genomic clones of *HrSEP-1* from the wild type and ‘Ryokusei’ and compared their gene structures. As shown in **Figure 6**, we detected a retrotransposon insertion in the first exon of *HrSEP-1* from ‘Ryokusei.’ BLAST analysis demonstrated that this retrotransposon insertion is a *Ty1/Copia*-like retrotransposon, which we named *Hret1*. PCR analysis using a primer set flanking to this insertion showed that ‘Ryokusei’ contains the mutant gene and not the wild type gene, indicating that the mutant allele of *HrSEP-1* containing *Hret1* is present in the homozygous state (**Figure 6**). Thus, the *Hret1* insertion likely leads to the formation of greenish flowers, and this mutation is likely to be recessive.

A functional analysis of *SEP*-like genes was previously carried out in *Phalaenopsis* using VIGS (Pan et al., 2014). The tepals of *PeSEP3-*silenced flowers with off-target silencing of *PeSEP1* and *PeSEP2* were converted into leaf-like organs, but column formation was not affected in these plants (Pan et al., 2014). The morphological differences in the columns of ‘Ryokusei’ vs. *PeSEP3-*silenced *Phalaenopsis* might have been due to residual activity of the *Phalaenopsis* *SEP3* ortholog. In other words, ‘Ryokusei’ showed the same phenotype as null mutants of *SEP3-*like genes, whereas residual expression of *PeSEP3* and *PeSEP1* might have helped maintain column formation in the *Phalaenopsis* VIGS line.

### HrSEP-1 Is Essential for Petal, Lip, and Column Development

In the perianths of ‘Ryokusei’ flowers, lips and petals were converted into sepaloid organs, whereas there were no morphological changes in sepals (**Figure 1**). These findings indicate that *HrSEP-1* plays an important role in the development of petals and lips, but not sepals. In ‘Ryokusei,’ the dorsal column was converted into sepaloid organs, indicating that *HrSEP-1* is also essential for column development (**Figure 1**). The dorsal column was converted into three to five sepaloid organs, suggesting that *HrSEP-1* likely functions in the transition of meristem activity from indeterminate to determinate growth in *H. radiata* flowers.

*Arabidopsis thaliana* contains four *SEP* genes whose functions are largely redundant (Pelaz et al., 2000, 2001; Ditta et al., 2004). On the other hand, in the current study, loss of function of *HrSEP-1* caused significant morphological changes in floral organs, even though *HrSEP-2* was highly expressed in the ‘Ryokusei’ mutant. These results indicate that *HrSEP-1* and *HrSEP-2* are not functionally redundant. In *Phalaenopsis*, downregulating *PeSEP3* by VIGS also had a significant effect on floral morphology (Pan et al., 2014). Meanwhile, silencing of *PeSEP2* had only minor effects on floral phenotype, even though the expression of *PeSEP2* was strongly downregulated in these plants. Therefore, although both *HrSEP-1* and *PeSEP3* belong to the SEP3 clade, as shown in **Figure 3**, orchid SEP3 and the SEP1/2/4 orthologs might not be functionally redundant.

According to the quartet model (Theissen and Saedler, 2001) in *A. thaliana*, the floral organ identity is specified by combinational protein interactions of ABCE-class MADS-domain transcription factors (Theissen, 2001). These quartets control gene expression by binding to the DNA of their target genes (Theissen, 2001). Sepal identity is determined by a complex of two A (AP1) class proteins and two E (SEP) class proteins, petal identity is controlled by a complex of one AP1 and one SEP protein together with one of each of the B class proteins APETALA3 (AP3) and PISTILLATA (PI), stamen identity is specified by a complex of one SEP, one AP3, one PI protein and the C (AG) class protein, and carpel identity is determined by a complex of two SEP proteins together with two AG proteins. Therefore, E class genes are essential for quaternary complexes. As shown in **Figure 6**, yeast two hybrid experiments demonstrated that HrSEP-1 interact with B and C class proteins. Suppressed expression of *HrSEP-1* gene in ‘Ryokusei’ is likely to affect the construction of quaternary complexes with SEP and B/C proteins, and this might be the cause of the greenish phenotype of this mutant.

In the ‘Ryokusei’ cultivar, B-, C-, and E-class genes were upregulated in the ventral column and in sepaloid organs that had been converted from the dorsal column in whorls 3 and 4 (**Figure 4**). If HrSEP-1 and other MADS-box genes interact via positive feedback loops, the expression levels of MADS proteins interacting with HrSEP-1 would likely decrease in the absence of HrSEP-1 function. In ‘Ryokusei,’ HrSEP-1 was not expressed, but B (HrDEF)-, C (HrAGs)-, and E (HrSEP-2)-class genes were upregulated compared with the wild type (**Figure 4**). Based on the studies in *A. thaliana*, SEP3 is involved in positive and negative cross-control of AG via complex formation. The AG/SEP3 complex undergoes positive autoregulation to coordinately regulate and maintain its own expression in whorls 3 and 4 (Gomez-Mena et al., 2005). SEU/LUG/SEP3 prevent AG transcription in all four floral whorls via negative regulation (Sridhar et al., 2006), but this negative regulation in the whorls 3 and 4 is antagonized by the activities of LFY, WUS and the positive autoregulatory AG/SEP3 complexes (Sridhar et al., 2006). Our expression results suggested that the upregulated expression of *HrAGs* in column of ‘Ryokusei’ may be caused by loss of negative control of SEU/LUG/SEP3.

According to the orchid code and P-code model, clade 3 *DEF*-like genes play an important role in lip development in orchid (Mondragón-Palomino and Theißen, 2011; Hsu et al., 2015). The clade 3 *DEF*-like gene is highly expressed in lips but is also weakly expressed in columns (Hsu et al., 2015). In ‘Ryokusei,’ the clade 3 *DEF*-like gene *HrDEF* was highly upregulated in sepaloid organs that had been converted from a dorsal column (**Figure 4**). Therefore, the homeotic conversion of the dorsal column to sepaloid organs in ‘Ryokusei’ is likely associated with the upregulated expression of *HrDEF* in the dorsal column. Meanwhile, there are no noticeable changes in sepal of ‘Ryokusei,’ even though the expression of *HrSEP-1* was downregulated. This indicates that *HrSEP-1* may not be necessary for sepal development in *H. radiata.* Another possibility is that other *SEP*-like gene which has redundant function of *HrSEP-1* might exist in *H. radiata*. Some orchids (e.g., *C. goeringii, Erycina Pusilla*, and *P. equestris*) have four *SEP*-like genes (Pan et al., 2014; Dirks-Mulder et al., 2017; Xiang et al., 2017), however, we have isolated only two *SEP*-like genes from *H. radiata*. So, there might be a few of undetected *SEP*-like genes in *H. radiata* and exhibit redundant functions in the floral organ identity.

Our findings demonstrate that the mutant phenotype of ‘Ryokusei’ is caused by the insertion of a retrotransposon in *HrSEP-1*. In addition, we demonstrated that this gene is essential for petal, lip, and column development and that it appears to function in floral meristem determination. ‘Ryokusei’ is a spontaneous mutant that was first found in Shodoshima Island in Japan. Since ‘Ryokusei’ is a homozygous mutant of *HrSEP-1*, this mutant might have been selected from self-pollinated progeny containing a heterozygous retrotransposon insertion. Although ‘Ryokusei’ is sterile, this cultivar can be maintained by vegetative (bulb) propagation, like other *H. radiata* cultivars. Since a null mutant of *SEP*-like genes has not yet been identified in orchid, ‘Ryokusei’ is highly important for further analyzing the role of SEP in orchid.

## Author Contributions

MM and AK: experimental design and manuscript preparation. MM: experiments. MM: data analysis. AK: supervision, funding, and reagents.

## Conflict of Interest Statement

The authors declare that the research was conducted in the absence of any commercial or financial relationships that could be construed as a potential conflict of interest.

## Supplementary Material

FIGURE S1 Phylogenetic tree of ‘Ryokusei’ and *Habenaria* species derived based on ITS sequences. Numbers on the nodes represent bootstrap values greater than 50% from 1000 replicates.

FIGURE S2 Expression analysis of *HrDEF, HrAG-1, HrAG-2, HrSEP-1*, and *HrSEP-*2 using semi-qRT-PCR. **(A)** Wild type. **(B)** ‘Ryokusei’. *eEF1A* was used as a positive control. Se, sepals; Pe, petals; Li, lip; Co, columns; Dco, dorsal column; Vco, ventral column.

TABLE S1 List of primers used in the present study.

TABLE S2 Accession numbers for the AG-like genes isolated in the present study and the genes used in the phylogenetic analysis.

TABLE S3 Accession numbers for the SEP-like genes isolated in the present study and the genes used in the phylogenetic analysis.
