# Supplementary figures and images for: The Greenish Flower Phenotype of Habenaria radiata (Orchidaceae) Is Caused by a Mutation in the SEPALLATA-Like MADS-Box Gene HrSEP-1
Source: Front Plant Sci. 2018 Jun 19;9:831. doi: 10.3389/fpls.2018.00831 (PMC6018480; doi:10.3389/fpls.2018.00831)

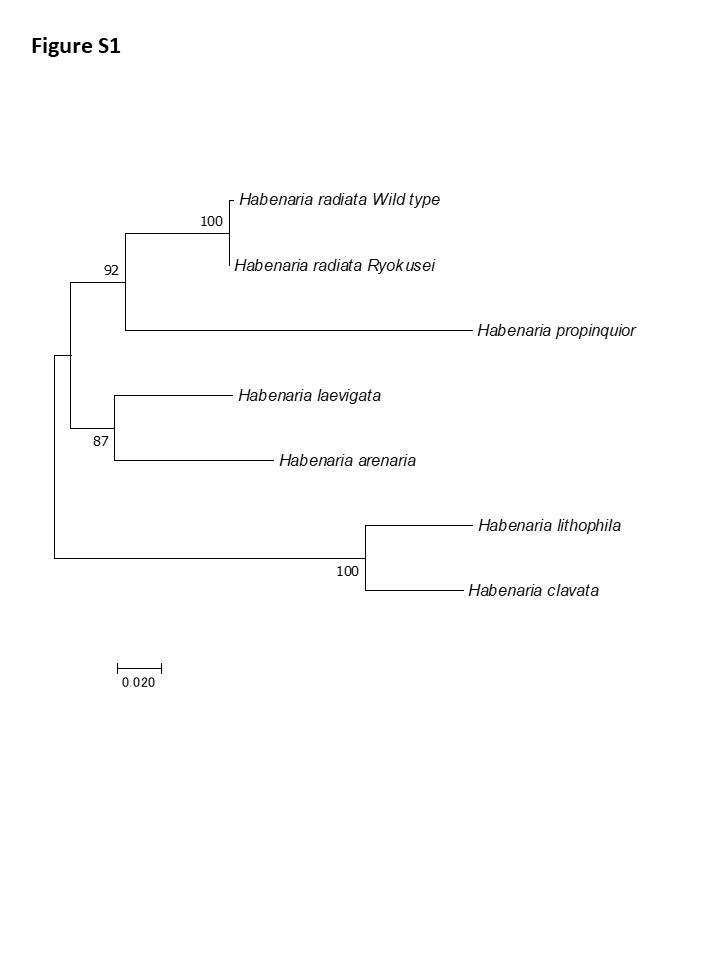

Supplement: FIGURE S1 — Phylogenetic tree of ‘Ryokusei’ and Habenaria species derived based on ITS sequences. Numbers on the nodes represent bootstrap values greater than 50% from 1000 replicates. [file Image_1.JPEG]

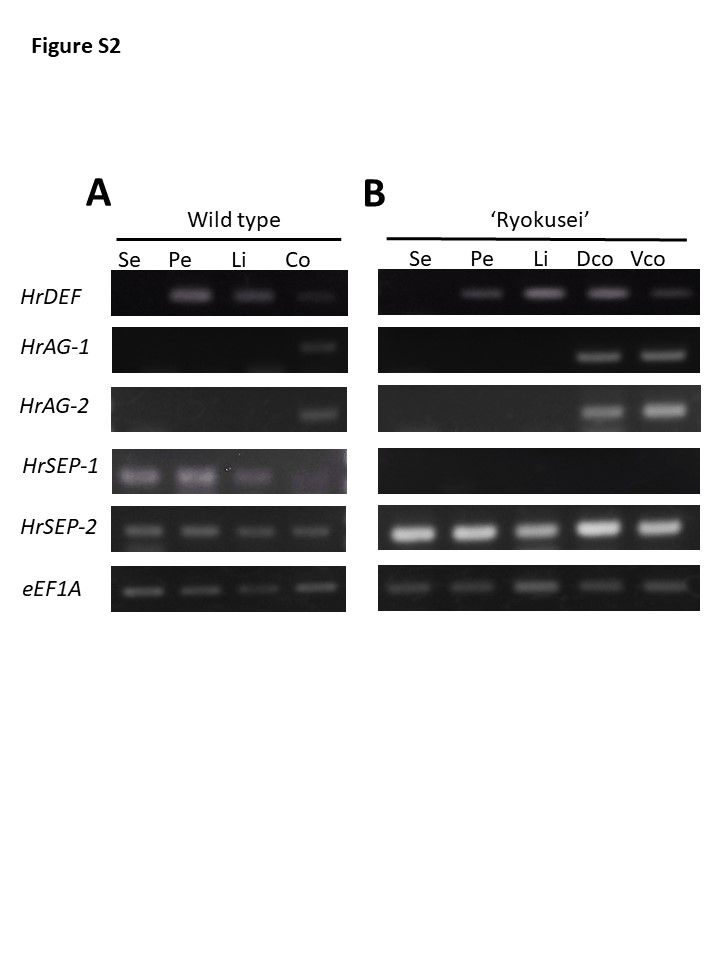

Supplement: FIGURE S2 — Expression analysis of HrDEF, HrAG-1, HrAG-2, HrSEP-1, and HrSEP-2 using semi-qRT-PCR. (A) Wild type. (B) ‘Ryokusei’. eEF1A was used as a positive control. Se, sepals; Pe, petals; Li, lip; Co, columns; Dco, dorsal column; Vco, ventral column. [file Image_2.JPEG]
